# Cardiac optogenetics: regulating brain states via the heart

**DOI:** 10.1038/s41392-023-01582-6

**Published:** 2023-08-30

**Authors:** Silvia Rodriguez-Rozada, Stefan Frantz, Philip Tovote

**Affiliations:** 1 Defense Circuits Lab, Institute of Clinical Neurobiology, University Hospital Würzburg, Würzburg, Germany; 2 Department of Internal Medicine I, University Hospital Würzburg, Würzburg, Germany; 3 Comprehensive Heart Failure Center, University Hospital Würzburg, Würzburg, Germany; 4 Center for Mental Health, University Hospital Würzburg, Würzburg, Germany

**Keywords:** Systems biology, Neuroscience, Cardiology, Neurology

**Fig. 1:**
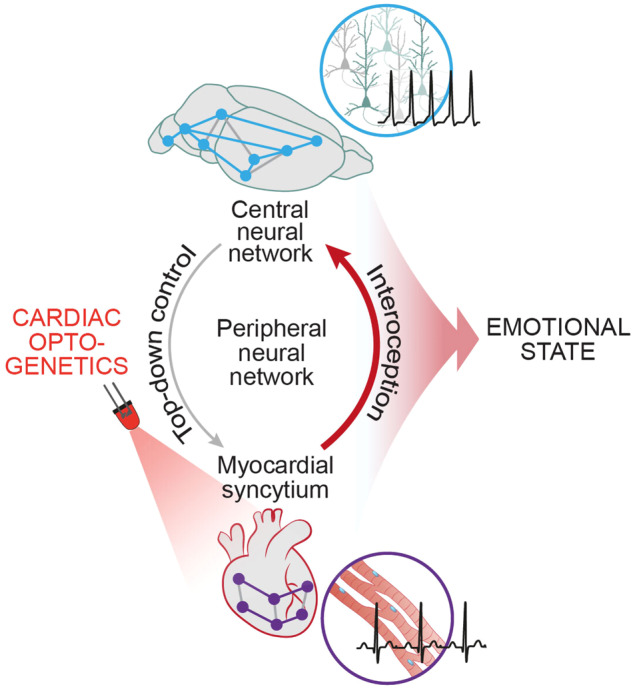
Cardiac optogenetics to affect brain activity. Schematic representation of circuitries connecting heart and brain. Central and peripheral parts of the autonomic nervous system play a central role in the neurogenic modulation of heart function. Hsueh et al. optogenetically activated cardiomyocytes, thereby causing tachycardia. This increase in heart rate, by way of interoceptive ascending information flow from the heart to the brain, led to increased neuronal activity in the posterior insular cortex. This was associated with increased anxiety-like behaviours in threatening contexts, reflecting enhancement of the aversive emotional state by higher heart rates

In a recent study published in *Nature*,^1^ Hsueh et al. developed a non-invasive optogenetic approach to control heart rate in freely moving mice. Optically induced tachycardia elicited an anxiety-like state in risky contexts, with the posterior insular cortex mediating the apprehensive behaviours arising from cardiac pacing (Fig. 1).

Whereas the link between emotions and bodily functions is part of the human experience, whether emotions are cause or consequence of their associated responses has been long debated by psychologists and neuroscientists. Does anxiety lead to an increased heart rate, or conversely, does a racing heart drive anxious behaviours? There is ample evidence linking mental and cardiac health. Clinical meta-analysis studies have shown an increased risk of heart disease in patients suffering from anxiety or stress-related disorders. Similarly, individuals with atrial fibrillation—a heart condition characterized by irregular and abnormal beating—have a higher prevalence of depression and its severity is associated with lower self-reported mental and physical health status.^2^ Although vagus nerve stimulation is clinically used to treat both conditions, little is known about the circuit mechanisms underlying heart-brain communication and how cardiac feedback to the brain, a process termed interoception, influences affective behavioural states.

The insular cortex is a key hub for interoceptive processing in the forebrain. It is involved in anxiety-related behaviours and maintains fear responses within a homeostatic range, for which it requires vagal input.^3^ Furthermore, specific defensive behaviours and heart rate dynamics have been shown to reflect context-dependent threat levels,^4^ thereby showcasing the intricate interplay between the heart and the brain in defining internal states. Nonetheless, the lack of tools for precise spatiotemporal modulation of heart function has hindered a detailed investigation of the mechanisms that regulate anxiety via interoceptive feedback.

Cardiac optogenetics allows light-mediated excitation of the heart with high spatiotemporal resolution and in a cell-type-specific manner. Optogenetic stimulation of cardiomyocytes has been successfully achieved in vitro and in anesthetized mice,^5^ enabling precise cardiac pacing with light. However, mostly owing to the technical difficulties of light-delivery in a contracting organ, cardiac optogenetics had not yet been applied in freely moving animals, a prerequisite that needs to be met to study the causal influence of heart dynamics on brain function and behaviour.

To overcome this challenge, Karl Deisseroth’s group took advantage of ChRmine, a red-light activated, highly sensitive pump-like channelrhodopsin previously used to modulate deep brain neuronal circuits without intracranial surgery. Delivery of light through the mouse intact chest using a wearable micro-LED vest was sufficient to stimulate ChRmine-expressing cardiomyocytes and to impose tachycardia in a non-invasive way. Optically increasing heart rate did not affect locomotion or pain perception, but mice showed reduced exploration and avoidance of risk-taking, behavioural changes that reflect anxiogenic-like effects in rodents. Furthermore, when a reward was coupled to a risk of mild electric foot shock, optically paced mice displayed decreased reward-seeking behaviour, which highlights the context-dependent enhancement of anxiety. Next, using activity-dependent neuronal labelling, whole-brain tissue clearing, and in vivo electrophysiology Hsueh et al. were able to correlate an increase in heart rate with increased neuronal activity in the posterior insular cortex, and subsequently confirmed the necessity of this brain region in mediating tachycardia-induced anxiety-related behaviours by optogenetic inhibition.

The work of Hsueh et al. opens up new possibilities to study heart-brain interactions. In addition to identifying brain regions that are specifically recruited by tachycardia and necessary for anxiety-related behaviours, their study marks a milestone in the field of cardiac optogenetics by developing a method for non-invasive precise control of heart rate in freely moving mice. The heart is a complex organ comprising different spatially and functionally interconnected populations of excitable and non-excitable cells which are tightly regulated to ensure correct cardiac performance. Moreover, cardiac activity is extrinsically modulated by heart-innervating autonomic neurons, which in turn, are orchestrated by higher-order neuronal circuits. Similar to the cell-type and pathway-specific strategies that are now widely used in systems neuroscience to dissect brain circuitry, optogenetic methods can shed light on cardiac network function and brain-heart communication.

Non-invasive cardiac optogenetic approaches hold a strong potential to overcome some of the limitations of existing methods for therapeutic heart stimulation. Compared to a classical electrical pacemaker, an optical pacemaker provides higher spatiotemporal precision and should therefore reduce unwanted side effects such as pain as well as pacing-induced stress in the patient. Nonetheless, the clinical applicability of optogenetic methods still faces major obstacles, including (1) the delivery of exogenous opsins by safe and sustainable AAV gene transfer strategies (2); phototoxicity and dysfunctional cellular reactions, especially during long-term stimulations; and (3) the development of implantable light devices that can adapt to cardiac movement while maintaining sufficient energy supply. Importantly, differences in organ dimension will require innovation of powerful, yet safe light emitters to achieve homogeneous and sufficient stimulation of cardiac tissue in humans. While gene and light delivery to the human heart comprise different constraints as compared to the eye, a promising clinical case of partial vision restoration after AAV-mediated optogenetic therapy was reported in 2021.^6^

A second implication derived from the work of Hsueh et al. is the possibility to modulate affective states by controlling bodily signals. Could heart rate be tuned in a specific manner to attenuate the pathological impact of psychiatric disorders such as anxiety or depression? By optogenetically stimulating cardiomyocytes Hsueh and colleagues could impose strong tachycardia, which caused enhanced anxiety-like behaviour. The opposite effect would be desirable to potentially treat emotional disorders associated with high sympathetic tone via downregulation of heart function and resulting interoception. In the long run, this could be achieved via specific optogenetic cardio-inhibitory approaches but in order to do so, we need a better understanding of how the heart conveys signals to the brain, and vice versa. Induction of tachycardia via stimulation of myocardial syncytium is a rather coarse manipulation of cardiac function and more specific stimulation approaches are required to fine-tune cardiac dynamics without interfering with the physiological beating function of the heart. This will open up new possibilities to explore the role of the heart-brain axis in psychiatric disorders. Hsueh et al. have achieved an important milestone for peripheral optogenetics in basic research, but have also paved the way for future clinical approaches targeting the peripheral nervous system for treatment of systems disorders.
